# Correlation Between Idiopathic Immune-Mediated Uveitis and Audiovestibular Involvement: A Cross-Sectional Study

**DOI:** 10.3390/jcm14103517

**Published:** 2025-05-17

**Authors:** Antonio Bustos-Merlo, Juana Dominguez-Perez, María del Carmen Olvera-Porcel, Antonio Espejo-González, Juan Manuel Espinosa-Sanchez, Nuria Navarrete-Navarrete

**Affiliations:** 1 Autoinmune and Systemic Diseases Unit, Department of Internal Medicine, Hospital Virgen de las Nieves, 18014 Granada, Spain; nuria.navarrete.sspa@juntadeandalucia.es; 2 Division of Otoneurology, Department of Otolaryngology, Hospital Virgen de las Nieves, 18014 Granada, Spain; juana.dominguez.perez.sspa@juntadeandalucia.es (J.D.-P.); juanmaespinosa@ugr.es (J.M.E.-S.); 3 Biostatistics, Unidad de Gestión y Apoyo a la Investigación, Hospital Virgen de las Nieves, 18014 Granada, Spain; mariac.olvera.sspa@juntadeandalucia.es; 4 Department of Ophthalmology, Hospital Universitario Virgen de las Nieves, 18014 Granada, Spain; antonio.espejo.sspa@juntadeandalucia.es; 5 Otology and Neurotology Group CTS495, Instituto de Investigación Biosanitaria ibs.GRANADA, 18012 Granada, Spain; 6 Division of Otolaryngology, Department of Surgery, University of Granada, 18071 Granada, Spain; 7 Sensorineural Pathology Programme, Centro de Investigación Biomédica en Red en Enfermedades Raras, CIBERER, 28029 Madrid, Spain; 8 MP03-Medicina Interna—HVN Investiga, Instituto de Investigación Biosanitaria ibs.GRANADA, 18012 Granada, Spain

**Keywords:** idiopathic immune-mediated uveitis, sensorineural hearing loss, vestibular disorders, vestibular tests, intraocular inflammation, multidisciplinary management

## Abstract

**Background/Objectives:** Idiopathic immune-mediated uveitis (IIMU) is an intraocular inflammatory condition affecting the uveal tract and adjacent ocular structures, potentially leading to systemic involvement. Audiovestibular symptoms, such as sensorineural hearing loss (SNHL) and balance disturbances, are often underdiagnosed in these patients. The potential correlation between IIMU and audiovestibular dysfunction remains insufficiently studied. This study aimed to estimate the prevalence and describe the clinical characteristics of audiovestibular manifestations in patients with IIMU. **Methods:** We conducted a cross-sectional observational study of 34 patients with a confirmed diagnosis of IIMU at a tertiary academic center. All participants underwent a standardized neurootological assessment, including pure-tone audiometry, video head impulse testing (vHIT), and cervical vestibular-evoked myogenic potentials (cVEMP). Demographic and clinical data were also collected. **Results:** Audiovestibular dysfunction was identified in 41.18% of patients, with bilateral SNHL (B-SNHL) being the most common finding. Patients with B-SNHL had a significantly later age of uveitis onset (52.3 ± 14.4 vs. 35.9 ± 13.9 years, *p* = 0.003) and a higher incidence of ocular complications (83.3% vs. 59.1%, *p* = 0.252). Furthermore, worsening ophthalmologic activity was observed in 25% of patients with B-SNHL, compared to 0% in those without B-SNHL (*p* = 0.037). Vestibular dysfunction was also associated with delayed onset of uveitis (51.0 ± 17.4 vs. 36.0 ± 12.2 years, *p* = 0.006) and a non-significantly higher complication rate (76.9% vs. 61.9%, *p* = 0.465). **Conclusions:** Audiovestibular dysfunction is a frequent finding in patients with IIMU and is associated with delayed uveitis onset and greater ocular morbidity. These results support the inclusion of systematic audiovestibular screening in clinical evaluations of IIMU patients and suggest that earlier detection may inform prognosis and guide multidisciplinary management strategies.

## 1. Introduction

Uveitis is an intraocular inflammatory disease that affects the uveal tract (iris, choroid, and ciliary body) and adjacent structures, including the sclera, cornea, vitreous humor, retina, and optic nerve head. It is the most common form of ocular inflammation, with an annual incidence of 52 cases per 100,000 individuals and a global prevalence of approximately 0.1% [1]. Uveitis predominantly affects individuals between 20 and 50 years of age (60–80%), with lower prevalence in children under 10 (7%) and adults over 70 (7.3%) [2]. This condition accounts for 10% of vision loss and contributes to 5–20% of legal blindness in developed countries [3]. Although the underlying pathogenesis remains poorly understood, uveitis is recognized as an immune-mediated disorder with contributions from endogenous and possibly environmental factors [4]. 

Classification systems now emphasize anatomical and etiological criteria [5]. Based on etiology, uveitis is grouped into infectious, systemic immune-mediated, ocular-specific syndromes, and masquerade syndromes (e.g., lymphoma, leukemia). Despite thorough diagnostic workups, 30–60% of cases remain idiopathic [6]. The term “idiopathic immune-mediated uveitis” (IIMU) designates non-infectious, non-neoplastic, non-traumatic, and non-systemic autoimmune uveitis that responds favorably to immunomodulatory therapy, including corticosteroids, immunosuppressants, and biologics. Typically, bilateral, chronic, or recurrent IIMU significantly impairs quality of life.

Traditionally regarded as an isolated ocular disorder, IIMU may also involve systemic immune-mediated manifestations, including audiovestibular dysfunction. Inner-ear disorders can produce a spectrum of auditory and vestibular symptoms, such as sensorineural hearing loss (SNHL), tinnitus, vertigo, imbalance, and aural fullness. These symptoms, although common, are often underdiagnosed in ophthalmology settings or misattributed to unrelated causes. Mismanagement may result in poor quality of life and secondary psychiatric symptoms [7]. The anatomical, embryological, and immunological similarities between ocular and inner-ear tissues suggest a potential shared immune-mediated mechanism. In recent years, interest in the immunopathogenesis of cochleovestibular disorders has grown [8].

Diagnosing immune-mediated inner-ear dysfunction remains challenging due to the absence of specific biomarkers [9]. Clinical suspicion, multidisciplinary collaboration, and early intervention are critical, as prompt treatment may prevent irreversible cochleovestibular damage [10].

**Figure 1:**
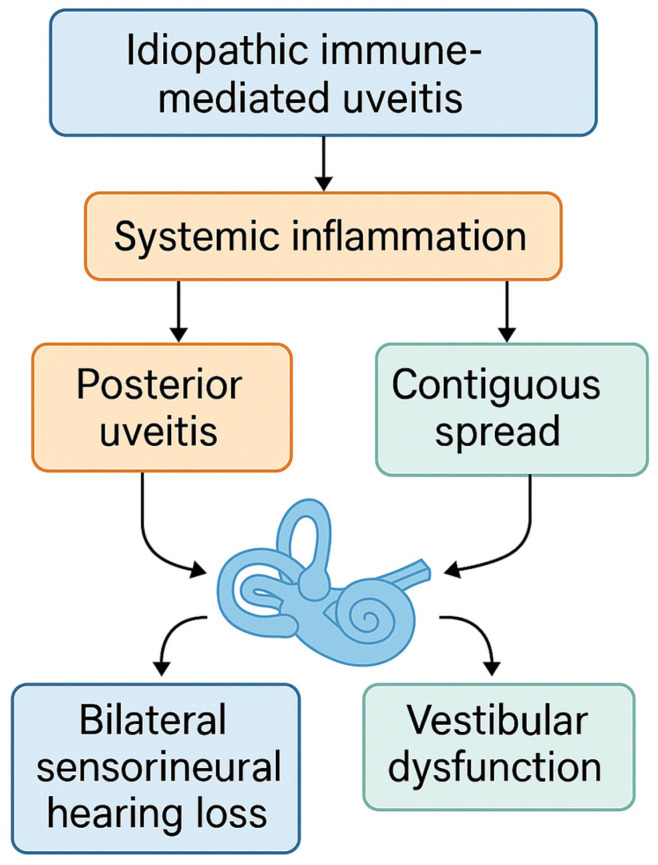
Proposed pathophysiologic model of audiovestibular involvement in IIMU.

Despite increasing recognition of immune-mediated cochleovestibular conditions, little is known about the prevalence and characteristics of audiovestibular involvement in IIMU. This cross-sectional observational study aims to investigate the prevalence of SNHL and vestibular dysfunction in patients with IIMU. We hypothesize that audiovestibular symptoms represent an extrapolation of the immune-mediated process beyond the uveal tract and that their presence is associated with delayed uveitis onset and increased ocular complications [Figure 1]. Early identification of these manifestations may facilitate timely systemic treatment and improve patient outcomes.

Systemic inflammation triggered by IIMU—especially in posterior uveitis—may lead to bilateral sensorineural hearing loss and vestibular dysfunction via contiguous or hematogenous spread to the inner ear.

## 2. Materials and Methods

### 2.1. Study Design and Population

This was a retrospective observational study conducted at the Uveitis Unit of the Granada North Health Area (Spain), covering a four-year period from January 2019 to December 2022. The aim was to evaluate audiovestibular manifestations in patients diagnosed with IIMU.

### 2.2. Participants

All adult patients (≥18 years) diagnosed with IIMU during the study period were considered for inclusion. IIMU was defined as non-infectious, non-neoplastic, and non-traumatic uveitis with no evidence of systemic autoimmune disease, and a favorable response to immunomodulatory therapy (corticosteroids, immunosuppressants, and/or biologics).

Inclusion criteria were as follows:

- A confirmed diagnosis of uveitis by a fellowship-trained ophthalmologist.
- Non-infectious, non-neoplastic, and non-systemic autoimmune etiology confirmed through comprehensive evaluation.
- Positive clinical response to immunomodulatory treatment.

Exclusion criteria included the following:

- Peripheral or central vestibular disorders with alternative diagnoses.
- Incomplete ophthalmologic and neurotological evaluation.

### 2.3. Data Collection and Clinical Assessment

Demographic variables, medical history, and relevant clinical findings were recorded. Demographic data, medical history, and clinical characteristics were extracted from electronic health records. Ophthalmologic examination included best-corrected visual acuity (BCVA), slit-lamp biomicroscopy, intraocular pressure measurement, and optical coherence tomography (OCT). Uveitis was categorized by anatomical localization (anterior or non-anterior: intermediate, posterior or panuveitis), laterality, onset (sudden or insidious), duration (limited or persistent), and course (acute, recurrent, or chronic), according to SUN (Standardization of Uveitis Nomenclature) criteria. Acute uveitis has sudden onset and lasts <3 months, recurrent uveitis involves episodes with ≥3 months of inactivity, and chronic uveitis persists with recurrences within 3 months after treatment discontinuation.

Deteriorating ophthalmologic activity was defined as any of the following: increased intraocular inflammation, reduction in BCVA attributable to inflammatory activity, or progression of uveitic complications over serial visits.

#### 2.3.1. Audivestibular Evaluation

All patients underwent a comprehensive audiovestibular assessment by neurotology specialists blinded to the ophthalmologic findings. The protocol included detailed medical history, neurotological examination, tympanometry, pure tone audiometry (PTA), video head impulse test (vHIT), and cervical vestibular-evoked myogenic potentials (cVEMP).

#### 2.3.2. PTA

Audiometric testing was performed using an Interacoustic AC40 audiometer (Intercoustics, Assens, Denmark) with TDH39-P supra-aural headphones (Telephonics, Farmingdale, NY, USA) inside a calibrated Sibelmed S-40 soundproof booth (Sibel, Sabadell, Spain). Air and bone conduction thresholds were measured from 125 to 8000 Hz. Masking was applied when appropriate. SNHL was defined according to the BIAP classification [11]. Normal corresponds to ≤20 dB, mild to 21–40 dB, moderate to 41–70 dB, severe to 71–90 dB, profound to 91–119 dB, and total or cochlear deafness to ≥120 dB.

#### 2.3.3. vHIT Assessment

vHIT was performed using ICS Impulse (GN Otometrics, Natus Medical, Taastrup, Denmark). Only horizontal semicircular canal function was assessed due to device limitations. Subjects fixated on a target 1 m away; 20 rapid, passive head impulses (15° to each side, 150–200°/s) were administered. Normal vestibulo-ocular reflex (VOR) gain was defined as 0.8–1.2, based on reference standards [12]. Abnormal vHIT indicates vestibulo-ocular reflex gain in at least one semicircular canal. The test was always performed by the same neurotologist to minimize interobserver variability. Tests that yielded inconclusive or non-physiological data were labeled as “non-evaluable” and included in the tables as missing values.

#### 2.3.4. cVEMP Evaluation

cVEMP testing was performed with Eclipse EP 25 (Intercoustics, Assens, Denmark) and insert earphones (IP-30 ABR, Radioear, Middelfart, Denmark). Surface electrodes (Ambu Neuroline 720, Ambu, Ballerup, Denmark) were positioned on the sternocleidomastoid muscle, sternoclavicular junction, and forehead (ground). Impedance was maintained at <5 kΩ (inter-electrode <3 kΩ). Patients sat upright and rotated their head contralaterally to ensure muscle activation. Tone bursts (500 Hz, 6 ms) were delivered at 100 dB nHL. Myogenic activity was maintained between 49.9 µV and 150.6 µV RMS, with artifact rejection ±800 µV. A response was considered absent if P1–N1 waves were not detected. The amplitude asymmetry ratio (AAR) was calculated as follows:

(AL − AS)/(AL + AS) × 100, where AL and AS denote the larger and smaller peak-to-peak amplitudes. cVEMP primarily evaluates the function of the saccule and the inferior vestibular nerve. Abnormal cVEMP was defined as absent response or AAR > 35%. Tests that yielded inconclusive or non-physiological data were labeled as “non-evaluable” and included in the tables as missing values.

### 2.4. Statistical Analysis

A descriptive analysis of the collected variables was performed, using measures of central tendency and dispersion for continuous variables and absolute and relative frequencies for categorical variables. The normality of continuous variables was assessed using the Shapiro–Wilk test.

To compare patients with and without bilateral sensorineural hearing loss (B-SNHL) and those with and without vestibular involvement, bivariate statistical tests were employed. Continuous variables were analyzed using the independent-samples *t*-test. Categorical variables were compared using Pearson’s chi-square test or Fisher’s exact test, as required. A *p*-value < 0.05 was considered statistically significant.

All statistical analyses were performed using STATA software, version 16.1 (StataCorp LLC, College Station, TX, USA).

## 3. Results

Demographics, clinical findings, characteristics of uveitis, and details regarding the neurotological examination of patients are listed in Table 1 and Table 2.

A total of 34 patients with IIMU were included, of whom 79.41% were women. The mean age at uveitis onset was 34 ± 15.99 years. Most cases presented with bilateral uveitis (85.29%), insidious onset (70.59%), and a persistent (89.24%) and chronic course (73.53%). A family history of SNHL was present in 23.53% of cases. Relevant systemic or neurological symptoms included headache (23.53%), subjective hearing loss (26.47%), vertigo (20.59%), and dizziness (20.59%). The most common cardiovascular risk factors were hypertension (38.24%) and smoking (23.53%). The predominant anatomical type of uveitis was non-anterior (64.70%). Antinuclear antibodies (ANAs) were positive in 44.12% of patients, with positivity defined as a titer of ≥1:160. Immunomodulatory treatments included corticosteroids (35.29%) and methotrexate (41.18%). At final follow-up, ophthalmologic activity had improved in 58.82% of cases and remained inactive in 26.47%, and ocular complications were present in 67.65%.

### Audiovestibular Assessment

PTA revealed SNHL in 41.18% of patients, to mild (29.41%), moderate (8.82%), and severe (2.94%) degrees. B-SNHL was the most frequent finding (35.29%), while unilateral hearing loss was observed in the right ear in 2.94% and in the left ear in 2.94%.

Abnormal results were identified in 14.71% of vHIT and 32.35% of cVEMP tests.

A comparison between patients with and without B-SNHL is shown in Table 3.

Patients with B-SNHL had a significantly later age at uveitis onset (52.33 ± 14.39 years) compared to those without B-SNHL (35.91 ± 13.92 years; *p* = 0.003). No statistically significant differences were found regarding sex distribution, family history, or cardiovascular risk factors.

Worsening ophthalmologic activity was significantly more frequent among patients with B-SNHL (25% vs. 0%; *p* = 0.037). Although ocular complications were more common in the B-SNHL group (83.33% vs. 59.09%), the difference did not reach statistical significance (*p* = 0.252).

A comparison between patients with and without vestibular dysfunction is shown in Table 4.

Vestibular dysfunction was also associated with significantly later uveitis onset (51.00 ± 17.41 years) compared to patients without vestibular involvement (35.95 ± 12.22 years; *p* = 0.006). No significant differences were observed in sex, family history, or cardiovascular risk factors.

Worsening ophthalmologic activity was more frequent in patients with vestibular dysfunction (15.38% vs. 4.67%; *p* = 0.544), as were ocular complications (76.92% vs. 61.90%; *p* = 0.465).

## 4. Discussion

This study highlights a high prevalence of audiovestibular manifestations among patients with IIMU, with over 40% exhibiting SNHL and more than one-third demonstrating vestibular dysfunction upon objective testing [13,14]. Notably, vHIT was limited to horizontal semicircular canals, which may underestimate the full extent of vestibular impairment. These findings are consistent with the previous literature on autoimmune uveitis [15,16] and systemic autoimmune disorders such as Vogt–Koyanagi–Harada and Cogan’s syndrome [17,18], supporting the hypothesis that shared immune-mediated mechanisms may affect both the eye and inner ear. In this context, IIMU should be considered a potentially systemic inflammatory condition with multi-organ involvement.

Clinically, audiovestibular dysfunction in IIMU was associated with a higher frequency of ocular complications and persistent inflammation. While not all associations reached statistical significance, the observed trends suggest potentially meaningful clinical correlations. This underscores the importance of early screening, interdisciplinary collaboration, and the timely initiation of immunosuppressive therapy.

A particularly relevant observation was the trend toward increased audiovestibular involvement in patients with non-anterior uveitis. Although the association was not statistically significant, it may reflect a broader or more systemic inflammatory phenotype. Prior research supports this hypothesis, suggesting that posterior segment inflammation may be more likely to affect the inner ear due to anatomical proximity and possible hematogenous dissemination [19,20,21]. 

Differential diagnoses must include infectious etiologies such as syphilis or tuberculosis, which may mimic immune-mediated uveitis with concurrent inner-ear involvement. While these were excluded in our cohort, their consideration remains crucial due to their distinct treatment implications.

Additionally, biologic therapies appeared to be associated with lower rates of audiovestibular dysfunction, supporting previous findings that early immunomodulation may preserve sensory function in autoimmune settings [22]. Although our study design precludes causal inference, this observation warrants further prospective evaluation. The nature of SNHL in autoimmune conditions has been described as fluctuating, progressive, or irreversible if left untreated [23]. While our cross-sectional design limited longitudinal evaluation, these data emphasize the need for early detection and management to prevent permanent deficits.

Interestingly, the low number of patients with severe SNHL may reflect referral or selection bias—patients with mild symptoms might be more likely to undergo ophthalmologic rather than otologic evaluation. Future studies incorporating systematic audiovestibular screening could yield more accurate prevalence estimates.

Overall, our results support the integration of routine audiovestibular assessment in the evaluation of patients with IIMU, particularly those with chronic or non-anterior forms. This may facilitate earlier intervention, reduce the risk of irreversible damage, and improve long-term functional outcomes.

## 5. Conclusions

This study emphasizes the importance of a multidisciplinary approach in the management of idiopathic immune-mediated uveitis. Audiovestibular dysfunction—particularly bilateral SNHL and vestibular abnormalities—is frequent in this population and may represent an underrecognized manifestation of systemic immune dysregulation. Periodic audiovestibular evaluation, especially in patients with non-anterior or chronic uveitis, should be considered part of standard care. Early identification enables timely, personalized treatment strategies, including immunosuppressive and potentially biologic therapies. These findings also advocate for the development of consensus diagnostic criteria for immune-mediated audiovestibular disease (IMAVD) and the inclusion of neurotological screening in future clinical guidelines.

### Limitations

This study has several limitations. First, the sample size was relatively small, limiting statistical power to detect some associations. Second, its cross-sectional design precludes the determination of temporal or causal relationships between uveitis and inner-ear dysfunction. Third, potential referral bias may have influenced the composition of the audiological cohort, with more symptomatic or complex cases more likely to be referred. The underreporting of vestibular symptoms could also lead to an underestimation of true prevalence.

The vestibular assessment was limited to horizontal canal vHIT and cVEMP, potentially overlooking dysfunction in vertical canals or utricle. Additional testing (e.g., vertical vHIT, caloric tests, oVEMP) would offer a more comprehensive assessment. Furthermore, the absence of standardized biomarkers for immune-mediated inner-ear disease complicates diagnosis and may contribute to underrecognition.

To address these limitations, future studies should employ prospective longitudinal designs and standardized audiovestibular protocols. This would enable the assessment of disease progression, treatment efficacy, and long-term outcomes. Despite these constraints, this study provides important insights into the interplay between uveitis and inner-ear involvement, reinforcing the need for integrated diagnostic and therapeutic approaches.

## Figures and Tables

**Table 1:** Demographics and clinical findings of patients with IIMU.

Demographics	
Age of onset, years **	34 ± 15.99
Female *	27 (79.41)
Family history *	
SNHL	8 (23.53)
Vertigo ^∆^	2 (5.88)
Tinnitus	2 (5.88)
Cardiovascular risk factors *	
Hypertension	13 (38.24)
DM2	4 (11.76)
Smoking	8 (23.53)
Hypercholesterolemia	6 (17.65)
Headache	8 (23.53)
Subjective hearing loss	9 (26.47)
Vertigo ^∆^	7 (20.59)
Dizziness	7 (20.59)
Laboratory parameters *	
ANA positivity	15 (44.12)
ENA positivity	3 (8.82)
ANCA positivity	0 (0)
APL positivity	1 (2.94)
IGRA positivity	2 (5.88)
ECA positivity	5 (14.71)

** Mean ± SD; * n (%); DM2: diabetes mellitus type 2; ANA: antinuclear antibodies; ENA: Extractable Nuclear Antigen; ANCA: Antineutrophil Cytoplasmic Antibodies; APL: Antiphospholipid Antibodies; IGRA: Interferon-Gamma Release Assays; ECA: Angiotensin-Converting Enzyme; HLA: Human Leukocyte Antigen. **^∆^** Vertigo ≥20 min rotational sensation, not position-induced (002E).

**Table 2:** Characteristics of uveitis and neurotological examination.

Characteristics of Uveitis *	
Non-anterior	22 (64.70)
Bilateral involvement	29 (85.29)
Insidious onset	24 (70.59)
Persistent course	30 (89.24)
Evolutionary course	
Acute	3 (8.82)
Recurrent	6 (17.65)
Chronic	25 (73.53)
Current treatment *	
Corticosteroids	12 (35.29)
Methotrexate	14 (41.18)
Mycophenolate mofetil	6 (17.65)
Azatioprine	6 (17.65)
Adalimumab	10(29.41)
Cyclosporine	1 (2.94)
Rituximab	2 (5.88)
Ophthalmologic activity assessment *	
Inactive	9 (26.47)
Worsening	3 (8.82)
Improvement	20 (58.82)
Remission	2 (5.88)
Ocular complications *	23 (67.65)
Neurotological examination*	
SNHL	14 (41.18)
Abnormal vHIT	5 (14.71)
missing	2 (5.88)
Abnormal cVEMPs	11 (32.35)
missing	2 (5.88)

* n (%); vHIT: video head impulse test; cVEMPs: cervical Vestibular Evoked Myogenic Potentials; dB HL: decibel hearing level.

**Table 3:** Comparison of clinical and demographic characteristics between patients with and without bilateral sensorineural hearing loss (B-SNHL).

	No B-SNHL (n = 22)	B-SNHL (n = 12)	*p*-Value
Age of onset, years **	35.91 ± 13.92	52.33 ± 14.39	0.003
Female *	17 (77.27)	10 (83.33)	1.000
Family history: SNHL *	6 (27.27)	2 (16.67)	0.681
Hypertension *	7 (31.82)	6 (50.00)	0.462
DM2 *	1 (4.55)	3 (25.00)	0.115
Smoking *	10 (45.45)	4 (33.33)	0.717
Hypercholesterolemia *	4 (18.18)	2 (16.17)	1.000
Non-anterior *	13 (59.09)	9 (75.00)	0.465
ANA positivity *	10 (45.45)	5 (41.67)	1.000
Severe uveitis (biologic drugs) *	9 (40.91)	3 (25.00)	0.465
Ophthalmologic activity: worsening *	0 (0.00)	3 (25.00)	0.037
Ocular complications *	13 (59.09)	10 (83.33)	0.252

** Mean ± SD; Student’s *t*-test was used. * n (%); Pearson’s chi-square test or Fisher’s exact test was used, as appropriate.

**Table 4:** Comparison of clinical and demographic characteristics between patients with and without vestibular impairment.

	No Vestibular Impairment (n = 21)	Vestibular Impairment (n = 13)	*p*-Value
Age of onset, years **	35.95 ± 12.22	51 ± 17.41	0.006
Female *	18 (85.71)	9 (69.23)	0.387
Family history: SNHL *	4 (19.05)	4 (30.77)	0.679
Hypertension *	7 (33.33)	6 (46.15)	0.491
DM2 *	2 (9.52)	2 (15.38)	0.627
Smoking *	9 (42.86)	5 (38.46)	1.000
Hypercholesterolemia *	4 (19.05)	2 (15.38)	1.000
Non-anterior *	13 (61.90)	9 (69.23)	0.727
ANA positivity ^∆^ *	10 (47.62)	5 (38.46)	0.728
Severe uveitis (biologic drugs) *	8 (38.10)	4 (30.77)	0.727
Ophthalmologic activity: worsening *	1 (4.67)	2 (15.38)	0.544
Ocular complications *	13 (61.90)	10 (76.92)	0.465

** Mean ± SD; Student’s *t*-test was used. * n (%); Pearson’s chi-square test or Fisher’s exact test was used, as appropriate. ^∆^ ANA positivity defined as titers ≥1:160.

## Data Availability

De-identified data are available on request preceded by a signed data access agreement form.

## Abbreviations

The following abbreviations are used in this manuscript:

AAR	Amplitude asymmetry ratio
ANA	Antinuclear antibodies
ANCA	Antineutrophil Cytoplasmic Antibodies
APL	Antiphospholipid Antibodies
B-SNHL	Bilateral sensorineural hearing loss
cVEMPS	Cervical vestibular-evoked myogenic potentials
DM2	Diabetes mellitus type 2
ECA	Angiotensin-Converting Enzyme
ENA	Extractable Nuclear Antigen
HLA	Human Leukocyte Antigen
IGRA	Interferon-Gamma Release Assays
IIMU	Idiopathic immune-mediated uveitis
SNHL	Sensorineural hearing loss
vHIT	Video head impulse testing
